# Against Offering “Faux Codes”: An Ethically Problematic Workaround

**DOI:** 10.1002/hast.70027

**Published:** 2025-12-11

**Authors:** Aimee Milliken, Richard Leiter

**Keywords:** faux code, end‐of‐life care, palliative care, deception, clinical ethics, moral distress, nursing, public trust in health care

## Abstract

*In another essay in this issue of the* Hasting Center Report, *an argument is made in favor of offering a “faux code” in the setting of a patient who cannot say no to her family who insists her code status remain “full.” Under the proposal, an unwritten agreement is made between the patient and the clinical team to withhold cardiopulmonary resuscitation while maintaining a “full code” status in the medical record. The ethical question in this case involves how best to support and uphold the patient's autonomy while minimizing the distress of the patient's family, and the “faux code” is proposed as an ethically supportable intervention. However, pressing ethical concerns remain. In this essay, we offer three main objections to the provision of a “faux code”: the role of deceit and its consequences for public trust, the impact on nurses and trainees, and the framing of autonomy under the faux code proposal*.

## Essay

In the essay “Offering ‘Faux Codes’: An Ethical Option for the Patient Who Can't Tell Their Family No,” Abram Brummett argues in favor of offering a “faux code” in the setting of a decisionally capacitated patient with metastatic cancer who, despite ongoing conversation, cannot say no to her family, who insists her code status remain “full.”^1^ The patient, Mrs. K, has repeatedly described her desire to avoid cardiopulmonary resuscitation (CPR) to the clinical team; however, each time she discusses this with her adult children, she acquiesces to their demand that she request CPR.

Under Brummett's proposal, the faux code would involve an agreement between the patient and the clinical team to withhold CPR in the event of a cardiac arrest while maintaining a “full code” status in the medical record. Ostensibly in the interest of supporting the patient's autonomy, Mrs. K would consent to a plan of care in which she and the team are in agreement that CPR will be withheld. The family, however, would be deceived into believing that the patient's code status remains full. This plan would not be documented in the medical record but would be passed along verbally between team members, even using veiled language or “surreptitious” communication if needed to ensure that the family does not discover the plan. A faux code in this context is differentiated from a slow code: in the latter, suboptimal or ineffective CPR may be offered to lead the family into believing that “everything” had been done, whereas in the former, no actual sham interventions are introduced. Here, we will distinguish between faux and slow codes as appropriate, given important distinctions between Brummett's proposal and the use of slow codes.

The ethical question facing the clinical team in this case, and cases like it, involves how best to support and uphold the patient's autonomy while also minimizing the distress of the patient's family. Brummett has argued that a faux code is an ethically supportable intervention in the context of this ethical question. Indeed, slow codes and their variations (sham codes, fake codes, time‐limited codes, and so on) have long been debated in the bioethics literature,^2^ and despite widespread objection to the practices, several authors have supported the slow code as an intervention of last resort.^3^ However, there are pressing ethical concerns that remain. The faux code raises some of the same concerns but is also troubling in some ways of its own. In what follows, we offer three main objections to the provision of a faux code: the role of deceit and its consequences for public trust, the impact on nurses and trainees, and the framing of autonomy under the faux code proposal.

## Deception and Public Trust

Fundamentally, the faux code involves an agreement between a patient and a team to engage in a plan meant to mislead the family, even though sham interventions are not part of the plan. This proposal is, by definition, deceitful. Indeed, in the proposed algorithm, lying to family members, albeit with indirect or “ambiguous” language, may even be recommended. Brummett suggests that clinicians may need to tell the family, “We plan to do all we can if she arrests.”^4^ The faux code thus undermines the clinicians’ ability to fulfill their fiduciary responsibilities of truth telling. Some authors, including Brummett, have argued that the avoidance of deception, and related veracity‐based obligations, can be overridden by competing considerations.^5^ However, Brummett's argument neglects to consider certain important downstream consequences—including the broader impact on public trust in the health care system. An endorsement of faux codes sets a concerning precedent, one likely to erode faith in health care broadly and, more specifically, one that is detrimental to the ethical climate of the particular institution. Moreover, in the (expected) event of the patient's death, the conflict may evolve to be the clinician's word (that the patient desired no CPR) against the family's (the patient agreed to a full‐code status), and the clinician's strategy will have already put them on the record as endorsing deceit.

There is growing understanding of the lasting impact of end‐of‐life care on the bereaved, including increased rates of psychological distress, anxiety, depression, and post‐traumatic stress disorder.^6^ Given the enduring impacts of end‐of‐life clinical decisions on families, Robert Truog, for example, has proposed that there are instances in which the interest of “surviving family members may take priority.”^7^ While we do not necessarily endorse this view, the faux code framework neglects to fully consider the family's lived experience. Not only are they now bereaved, but they are also deceived—a combination that, should they discover the deception, is likely to prolong the lasting effects of a traumatic end‐of‐life experience. Inasmuch as patients are part of family units, the faux code framework violates the principle of nonmaleficence by inflicting harm on the family. Furthermore, even when patients disagree with their loved ones or surrogates, patients rarely, if ever, wish ill upon them. We doubt patients would agree to a faux code if, as would be necessary to achieve a fully informed consent, clinicians adequately explained the potential downstream consequences to them.

One could perhaps turn to public‐preference data to attempt to justify the faux code as an intervention and to argue that the impact on the family may not be as egregious as described here. For example, Phillip Sprengholz demonstrates some degree of public support for a slow code^8^—during which CPR and related interventions are offered, albeit at a slower and less effective pace. Given these important distinctions, however, it is not actually clear that public support for the slow code would extend to public support of the faux code, under which medical staff would offer *no* interventions and would be actively, intentionally deceiving the family. Public‐perception data about deception in medicine is far less equivocal—with a majority of respondents disapproving of deception if it involves outright lying.^9^ It is likely that public support of the slow code reflects the well‐documented public misunderstanding of the efficacy of CPR^10^ and the misplaced belief that a team offering *something* is at least better than the team offering *nothing*.

Indeed, a recent argument in favor of offering a slow code involves the conflictual health care environment and the increasing animosity experienced between clinical teams and families at the point of care.^11^ One could imagine extending this reasoning to the provision of a faux code. On this view, the slow code and faux code are akin to workarounds: if clinicians cannot fix widespread public division, they can at least avoid worsening conflict in the case in front of them by attempting to appease the unhappy or litigious surrogate, either by offering a sham intervention (in a slow code) or by pretending to agree to a potential intervention they plan never to provide (in a faux code). However, given the pivotal role of deception within the faux code, it is likely that, the more public knowledge about the practice grows, the worse public trust in the health care system would become. In this way, the faux code would actually serve to worsen a dynamic it might be employed to mitigate. This deleterious impact on public trust will be felt especially acutely by those who have historically been mistreated by the system—who already do not believe that they will receive the best care or be treated equitably and honestly—and, in particular, among marginalized communities who are more likely to request intensive life‐sustaining measures at the end of life.^12^

## Impact on Nurses, Trainees, and Other Allied Health Professionals

A second concern about the faux code involves the impact on nurses, trainees, and other allied health professionals. Each of these groups will be tasked with implementing the plan, yet members of these groups typically lack the decisional authority to independently agree to the proposed intervention in the way that an attending physician does. Even if the attending physician is in full support of the faux code and all that it entails, it does not follow that all team members will be as well.

Nurses, in particular, are at the bedside in a prolonged and embedded capacity, typically serving as the first point of communication between the patient or family and the rest of the clinical team. Nurses are thus likely to be the ones fielding the majority of questions at the bedside from family members around the clock. In addition, it is often the nurse who identifies a cardiac arrest and initiates resuscitative efforts, including compressions. In many instances, the nurse would be tasked with initiating the faux code. In addition to the communication concerns, the faux code proposal may have problematic impacts on nursing licensure. If the documented code status is “full” but the attending physician verbally tells the nurse to withhold CPR without supporting documentation, this creates a dilemma: should the nurse adhere to the medical record and protect their license or abide by the requests of the attending?

One could argue that survey data about slow codes suggest that nurses may actually support the provision of a faux code and view the practice as ethical. However, it is important to view the research on slow codes in context. For example, Gina Piscitello and colleagues found that a majority of nurses surveyed view a slow code as ethically acceptable “if the patient or alternate decision maker requests CPR,” with the caveat that “[t]he alternate decision maker should be notified that the CPR duration will be limited.” Support for this practice drops dramatically when the caveat that the surrogate is not notified is added to the question.^13^

Under the faux code framework, the surrogate decision‐maker is not informed, and the nurse is asked to deceive or actively lie to them. It is well documented that the inability to uphold professional obligations, such as truth telling, is a contributor to moral distress.^14^ This obligation is explicated in the 2025 American Nurses Association Code of Ethics, which serves as the discipline's nonnegotiable ethical standard. The Code of Ethics defines the nurse's fiduciary duties as being inclusive of “truthfulness, promise‐keeping, and dedication in relationships.”^15^ Nurses are perennially viewed as the most trusted of the helping professions^16^ and typically deeply value their veracity‐based obligations as a matter of professional identity.^17^ It therefore stands to reason that, when the full extent of potential deceit is described, any support of the intervention would waver or crumble. The faux code would likely contribute to distress for nurses and other clinicians at the point of care, who may feel that their agency is constrained and that they are active participants in a plan of care that stands counter to their professional ethical obligations^18^—a situation that is a recognized source of moral distress and related sequelae, including burnout and attrition.^19^

Trainees merit special attention here. Often working as responding clinicians and code team leaders, particularly overnight, trainees serve as the effectors of code status orders but frequently lack decision‐making authority in code‐status discussions and decisions. If trainees were assigned to care for patients with a faux code status, this combination of responsibility and lack of authority would position them on a clear path toward moral distress and moral injury. Instructing trainees to follow this code status would also set a dangerous pedagogical precedent—that truth telling can and should be forsaken in challenging situations.

Even definitional nuance—slow code versus faux code versus sham code, for example^20^—can lead to inconsistencies or misunderstandings at the point of care, contributing to distress. Should the code not be called right away? Should compressions be started? Slowly? Shallowly? Adequately but for a briefer duration? Should we push resuscitative medications, or not? Should the family be at the bedside, and what should they be told? When do we lie to them, and what if they challenge us? What if the family tries to intervene? The actual implementation of the plan and its minutiae falls to those at the bedside—nurses and trainees—who must then deal with the immediate consequences of these decisions. To assume that the attending physician can be the sole individual to shoulder the burden of implementation and communication within the context of the faux code proposal ignores the realities of clinical care.

The degree of deception involved in carrying out a faux code would require interdisciplinary coordination on a complex and intricate scale, despite what Brummett argues about faux codes and what has been argued about coordination for slow codes.^21^ Indeed, the standard mechanisms that hospitals have for communicating information across shifts and between clinical teams are notoriously inadequate.^22^ Effectuating such a plan with only verbal handoff shift to shift (to avoid a paper trail visible to the family in the medical record) would create confusion and significant communication breakdown. For example, notes from family meetings and ethics consultations are often neither seen nor read by all members of the clinical team, particularly as time elapses. This leads to an evolving narrative as clinicians rotate and shifts change; understandings reached by teams and families earlier in the clinical course may not seem as clear as time goes on.

To mitigate this concern and to avoid having the plan discovered by the family, Brummett suggests minimizing verbal communication in the room about the patient's faux code status and relying on signaling (with a colored zip tie on a piece of bedside equipment, for example) or other less‐visible strategies. Not only does this approach increase the discrepancy between what is in the medical record and what has been agreed upon by the interdisciplinary team, but it also creates an additional hierarchical challenge regarding the implementation of the plan: on whose authority is CPR to be withheld? Historically, practices such as this, including the La Guardia Hospital's purple dot system in the 1980s, which was used to indicate patients from whom CPR was to be withheld without creating a paper trail in the medical record,^23^ have been described as “horrible procedural abuses” and have set medical ethics and serious illness communication back decades, including opening hospitals up to legal investigations. Even if one grants deception as defensible in this context (which we do not), no plan can predict every eventuality, and the catchall for these gaps, again, will fall to those at the bedside for the greatest duration. This prospect is likely to cause anxiety, particularly shift to shift, as nurses, trainees, and other clinicians learn of the proposal without necessarily having the benefit of participating in the conversations in which it was agreed to.

## Framing of Autonomy: Acquiescence to Family as an Extension of Autonomy

Our final and perhaps most critical objection to the faux code framework is about how it conceptualizes patient autonomy. For it to be ethically defensible, one must conceive of the patient as consenting to DNR and objecting to being a full code. However, as described in the case of Mrs. K, the patient herself has acquiesced to familial pressure and reversed her code status. Assuming the patient is capacitated, which under this proposal she must be, this acquiescence can actually be viewed as an extension of her autonomy; she has changed her mind to avoid conflict and to appease her children. Indeed, this pattern of decision‐making has likely been repeated many times throughout this patient's life course—a parent putting her own interests aside to support or please her children. We see this time after time in clinical care: the patient who agrees to another round of chemotherapy because his children are not ready to lose him or the patient who continues dialysis to stay alive for her daughter's wedding. While Mrs. K's decision may stand counter to the recommendations of the clinical team, it remains a decision that is within her purview.

It may be, and perhaps it is likely, that the patient wishes she had not agreed to reverse her code status. Indeed, in the case description, Mrs. K would become regretful when speaking in private with the clinical team. In this instance, it is the obligation of the team to support the patient either (a) in upholding her choice to change her code status to DNR and in affirming this decision to the family with compassionate boundaries or (b) through understanding her decision to acquiesce to the family as being reflective of a choice by which she has prioritized the interests of her children over her own. Indeed, the temptation may be to “fix” the disagreement at hand through the provision of a faux code. However, it is not the role of the clinical team to either predict or control the family's response to the provision of care that is both appropriate *and* requested by the patient. This is not to say that her decision cannot change—ongoing conversation between the team, patient, and family may help the patient elucidate her core concerns and help her family come to terms with her clinical decline and impending death. It may also help Mrs. K gain the confidence to make a decision she can defend, in concert with a supportive clinical team. If CPR in this case is indeed “not morally indicated,” then it ought to fall upon the team to transparently uphold this boundary. Clinicians cannot, however, agree to offer “bad” or deceitful care simply because it has been demanded or feels like the less conflictual way out.

Palliative care may play a critical role in such situations and allow primary teams to more effectively engage with the conflict. Palliative care clinicians hold expertise in serious illness communication, which has been shown to decrease anxiety and improve patient‐ and family‐centered outcomes.^24^ These skilled conversations can often uncouple anticipatory grief from what seem to be “unreasonable” requests for intensive life‐sustaining treatments. By using deceit to ostensibly acquiesce to the adult children's request, clinicians may, in fact, be robbing them of an opportunity for difficult yet emotionally important conversations with their mother at the end of life. To be sure, not all intrafamilial conflicts around code status can be mitigated by skilled serious illness communication, but many tensions or conflicts can be defused.

Many have argued that, in an effort to correct for paternalism, the pendulum of autonomy has swung too far and that clinicians have falsely given families the impression that they are in the position to make clinical determinations such as whether CPR is medically appropriate and should be offered.^25^ That problem, however, is not solved by the provision of a deceitful intervention. The solution involves empowering the patient by empowering the clinical team. In this way, the team can support the patient's autonomy unyieldingly yet compassionately.
